# Cisatracurium in different doses versus atracurium during general anesthesia for abdominal surgery

**DOI:** 10.4103/1658-354X.71571

**Published:** 2010

**Authors:** A. M. El-Kasaby, H. M. Atef, A. M. Helmy, M. Abo El-Nasr

**Affiliations:** *Department of Anaesthesia, Faculty of Medicine, Suez Canal University, Ismailia, Egypt*

**Keywords:** *Cisatracurium*, *atracurium*, *different ED95 dose and neuromuscular monitoring*

## Abstract

**Background:**

Cisatracurium in clinical practice is devoid of histamine-induced cardiovascular effects. On the other hand, 2 ED_95_ doses of cisatracurium (100 µ g/kg) do not create satisfactory intubating conditions such as those seen with equipotent doses of atracurium. The recommended intubating dose of cisatracurium is 3 ED_95_. To understand this discrepancy better, we evaluated the potency and onset of atracurium and cisatracurium.

**Materials and Methods:**

The study designed as randomized controlled clinical trial to compare between atracurium (2×ED_95_) and different doses of cisatracurium (2×ED_95_, 4×ED_95_, 6×ED_95_) regarding onset time, duration of action, condition of intubation, hemodynamic effects, and sings of histamine release clinically. Sixty four patients were randomly assigned to one of four groups, the first group (group 1) received 2×ED_95_ dose of atracurium, group 2 received 2×ED_95_ dose of cisatracurium, group 3 received 4×ED_95_ dose of cisatracurium, while group 4 received 6×ED_95_ dose of cisatracurium. The Datex relaxograph (Type NMT-100-23-01, S/N: 37541) for neuromuscular monitoring was used.

**Results:**

HR, MABP was statistically significant increased post-intubation with administration of 2×ED_95_ dose of atracurium in group 1 and the same dose of cisatracurium in group 2 but 5-20 min later was not statistically significant with administration of 4×ED_95_ and 6×ED_95_ doses of cisatracurium in groups 3 and 4, respectively. Onset time was found to be significantly lower with 2×ED_95_ dose of atracurium than with the same dose of cisatracurium. At the same time, higher doses of cisatracurium (4×ED_95_ and 6×ED_95_) showed onset time and longer duration of action that was significantly lower than with atracurium and with lower dose of cisatracurium (2×ED_95_). Only 6×ED_95_ dose of cisatracurium showed statistically significant difference versus the atracurium dose with higher percentages of patients with excellent condition of intubation. 4×ED_95_ and 6×ED_95_ doses of cisatracurium were significantly better than 2×ED_95_ dose of cisatracurium. 2×ED_95_ dose of atracurium and 2×ED_95_ dose of cisatracurium were similar, while 4×ED_95_ and 6×ED_95_ doses of cisatracurium were significantly better than atracurium and 2×ED_95_ dose of cisatracurium.

**Conclusion:**

The same dose (2×ED_95_ dose) atracurium is more effective neuromuscular blocking agent than cisatracurium, while higher doses of cisatracurium 4×ED_95_ and 6×ED_95_ provide more effective, more rapid neuromuscular blocking with longer duration of action, stable hemodynamic status, and no associated signs of histamine release clinically.

## INTRODUCTION

Muscle relaxants rapidly became a routine part of the anesthesiologist’s drug arsenal.[1]

The neuromuscular blocking potency of cisatracurium (NIMBEX) is approximately three-fold that of atracurium besylate, the time to maximum block is up to 2 min longer for equipotent doses of NIMBEX compared to atracurium besylate. The clinically effective duration of action and rate of spontaneous recovery from equipotent doses of NIMBEX and atracurium besylate are similar.[2]

Although cisatracurium is more potent than the parent mixture (95% effective dose (ED_95_) 0.05 mg/kg vs. 0.2 mg/kg), its pharmacodynamic profile is similar to that of atracurium, except for a reportedly slower onset.[3] Cisatracurium unlike atracurium is devoid of histamine-induced cardiovascular effects. On the other hand, 2 ED_95_ doses of cisatracurium (100 μg/kg) do not yield satisfactory intubating conditions such as those seen with equipotent doses of atracurium. The recommended intubating dose of cisatracurium is 3 ED_95_.[4,5]

### Aim of the work

This study was designed to compare between atracurium (2×ED_95_) and different doses of cisatracurium (2×ED_95_, 4×ED_95_, 6×ED_95_) regarding onset time, condition of intubation, duration of action, hemodynamic effects, and signs of histamine release.

## MATERIALS AND METHODS

The study was carried out as a comparative clinical trial on patients of both sexes underwent elective abdominal surgery in Suez Canal University Hospital in the routine surgical theaters.

### Inclusion criteria

ASA I and ASA II patients aged from 20 to 65 years old, both sexes scheduled for abdominal surgeries of an anticipated duration of at least 1 h and half included in the study. Exclusion criteria were any disorder of the cardiovascular, hepatic, renal, or neuromuscular systems known from history or clinical examination. Patients in whom difficult intubation was expected: pregnant or lactating women and patients on medication known to interact with neuromuscular blocking drugs e.g. Antibiotics (aminoglycosides and tetracycline), antidepressants, anticonvulsants antiarrhythmics (calcium channel blockers and quinidine) and magnesium sulfate. The detectable difference between the means of the group using the onset of action (time from end of injection to 90% neuromuscular block) and it equals 0.7 minutes.[6]

The calculated sample is 16 per group with total sample size 64 for the four groups of the study.

The 64 patients were equally and randomly divided into four groups:

Group 1: atracurium for 16 patients with initial dose of 0.5 mg/kg (2×ED_95_).
Group 2: cisatracurium for 16 patients with initial dose of 0.1 mg/kg (2×ED_95_).
Group 3: cisatracurium for 16 patients with initial dose of 0.2 mg/kg (4×ED_95_).
Group 4: cisatracurium for 16 patients with initial dose of 0.3 mg/kg (6×ED_95_). Patients were randomly allocated using an online research randomizer (http://www.randomizer.org/) into four equal groups.

The techniques were explained to patients including benefits and complications of each and written consent was taken. The patient’s age, sex, ASA status, duration, and type of surgery were recorded. Patients were premedicated using 2 mg midazolam through IV route 20 min preoperatively.

Monitoring equipments (Datex-Ohmeda^™^) were attached to the patient including three leads ECG, non-invasive blood pressure, pulse oximetry, capnography, and temperature probe. The Datex relaxograph (Type-NMT-100-23-01, S/N: 37541) for neuromuscular monitoring. The first response (T_1_) of the train of four (TOF) stimulation was the parameter, which used for the pharmacodynamic measurements. The hand, wrist and half of the forearm were wrapped with crepe bandage to avoid hypothermia. Patients were preoxygenated with 100% oxygen for 3 min.

General anesthesia was induced with fentanyl (1-1.5 μg/kg), followed 20 s later by propofol (2 mg/kg) intravenously. Anesthesia was maintained with a mixture of 50% N_2_O in O_2_ and isoflurane (0.5%-1.5% vol%) and assisted ventilation. Neuromuscular monitoring was carried out after obtaining the control values by supramaximal stimulus (70 mA) from relaxograph (2 Hz/0.5 s; pulse width 0.2 ms) every 20s to stimulate the ulnar nerve via surface electrodes.

After a stable base line period of at least 5 min, the muscle relaxant was given for patients according to the previously mentioned initial doses for each group and injected intravenously within 5-10 s. After 2 min, endotracheal intubation was attempted using proper size tube (male: 8-9, female: 7-7.5) and the condition of intubation was assessed and recorded according to the following:[7]

1. Excellent: Easy passage of the tube without coughing. Vocal cords relaxed and abducted.
2. Good: Passage of the tube with slight coughing and/or bucking. Vocal cords relaxed and abducted.
3. Poor: Passage of tubes with moderate coughing and/or bucking vocal cords moderately adducted.
4. Not possible: Vocal cords not relaxed, tightly adducted.

The onset time was determined as the interval from the end of muscle relaxant injection until the maximal suppression of T_1_%.

Anesthesia was maintained with a mixture of 50% N_2_O in O_2_, isoflourane (1-1.5 MAC), boluses of the muscle relaxant (10% of the initial dose) with 25% recovery of response to T_1_% and ventilation was controlled by the Datex-Ohmeda^™^ ventilator which will adjust end tidal CO_2_ at (30-35 mmHg). Neuromuscular blockade after induction was monitored and recorded every 5 min by supramaximal train-of-four (TOF) stimuli.

The duration of the muscle relaxant (time from the end of injection of the drug until 25% recovery of T_1_%) was recorded.

Patients were monitored for any signs of histamine release clinically through skin changes graded as flush (if redness lasted> 120 s), erythema, or wheals[8] and presence of any hemodynamic changes or bronchospasm.

Intra-operative hemodynamic changes were continuously displayed on the monitor including: heart rate (HR), mean arterial blood pressure (MABP) every 5 min, oxygen saturation (SO_2_), and end tidal CO_2_.

Body temperature was maintained between 35 and 37°C by means of warmed IV fluids and warming blankets (body core temperature through nasopharyngeal probe and skin temperature probe).

At the end of operation with 25% recovery of T_1_%, reversal (induced recovery) was achieved by administration of neostigmine and atropine mixture (2.5 mg neostigmine: 1 mg atropine) through slow IV injection. TOF-ratio>0.9 was sufficient for safe extubation of the trachea.

### Statistical methods

Data were processed using SPSS version 15 (SPSS Inc., Chicago, IL, USA). Quantitative data were expressed as means±SD while qualitative data were expressed as numbers and percentages (%). Student *t* test and ANOVA test were used to test significance of difference for quantitative variables (HR, BP) that follow normal distribution and chi square was used to test the significance of difference for qualitative variables. A probability value (*P*-value)<0.05 was considered statistically significant.

## RESULTS

The studied patients were matched regarding age and sex with no statistically significant difference being recorded [Table 1].

**Table 1:** Demographic characteristics of the studied patients

			Atracurium group (n=16) 2×ED_95_	Cisatracurium group (n=16) 2×ED_95_	Cisatracurium group (n=16) 4×ED_95_	Cisatracurium group (n=16) 6×ED_95_	*P*-value
Age	Mean ± SD		43.3 ± 6.5	45.7 ± 3.1	42.9 ± 7.3	39.9 ± 5.4	0.05
	Range		(28 – 54)	(31 – 56)	(27 – 50)	(29 – 53)	(NS)
Sex	Male	N (%)	9 (56.25%)	8 (50%)	6 (37.5%)	7 (43.75%)	0.7
	Female	N (%)	7 (43.75%)	8 (50%)	10 (62.5%)	9 (56.25%)	(NS)

NS - No statistically significant difference

There was a statistically significant increase in HR, MABP post intubation when compared to baseline and postinjection of 2×ED_95_ dose of atracurium in group 1 and the same dose of cisatracurium in group 2. HR, MABP changes 5-20 minutes later were not statistically significant with administration of 4×ED_95_ and 6×ED_95_ doses of cisatracurium in groups 3 and 4, respectively [Tables 2 and 3].

**Table 2:** Heart rate changes before and after administration of atracurium or cisatracurium

	Heart rate (beat/min)						
	Baseline reading	After injection of muscle relaxant	After attempt of intubation	5 min	10 min	15 min	20 min
Atracurium group (n=16) 2×ED_95_	69.8 ± 6.65	73.8 ± 7.9	83.1 ± 7.96 *	73.9 ± 8.96	73.3 ± 6.81	74.1 ± 6.75	73.6 ± 6.57
Cisatracurium group (n=16) 2×ED_95_	73.4 ± 5.61	73.9 ± 6.3	84.6 ± 547 *	76.3 ± 6.51	75.4 ± 5.87	73.6 ± 5.78	74.1 ± 5.65
Cisatracurium group (n=16) 4×ED_95_	70.8 ± 4.83	71.2 ± 5.2	75.2 ± 3.45	74.9 ± 6.53	76.7 ± 5.56	73.1 ± 6.75	72.6 ± 5.84
Cisatracurium group (n=16) 6×ED_95_	71.1 ± 6.54	71.8 ± 7.3	74.9 ± 5.54	73.8 ± 6.45	75.4 ± 6.85	72.4 ± 4.75	73.7 ± 5.38

* Statistically significant difference versus Baseline reading (*P*-value < 0.05)

**Table 3:** Mean arterial blood pressure changes before and after administration of atracurium or cisatracurium

	Mean arterial blood pressure (mmHg)						
	Baseline reading	After injection of muscle relaxant	After attempt of intubation	5 min	10 min	15 min	20 min
Atracurium group (n=16) 2×ED_95_	81.5 ± 8.37	78.9 ± 9.4	91.6 ± 6.4 *	86.4 ± 7.28	82.6 ± 6.89	81.5 ± 8.75	80.7 ± 7.54
Cisatracurium group (n=16) 2×ED_95_	80.9 ± 10.7	79.8 ± 10.5	92.6 ± 7.59 *	88.4 ± 8.64	84.6 ± 9.65	85.7 ± 7.43	83.5 ± 8.93
Cisatracurium group (n=16) 4×ED_95_	82.6 ± 9.5	82.1 ± 8.5	84.5 ± 6.73	83.5 ± 7.52	85.6 ± 8.42	84.9 ± 8.64	86.4 ± 9.24
Cisatracurium group (n=16) 6×ED_95_	81.7 ± 10.1	80.1 ± 9.6	85.3 ± 8.43	86.4 ± 9.34	84.9 ± 6.95	85.9 ± 6.58	82.6 ± 8.68

* Statistically significant difference versus Baseline reading (*P*-value < 0.05)

Time onset was found to be significantly lower with 2×ED_95_ dose of atracurium than with the same dose of cisatracurium. At the same time, higher doses of cisatracurium (4×ED_95_ and 6×ED_95_) showed onset time that was significantly lower than with atracurium and with lower dose of cisatracurium (2×ED_95_). Regarding the duration of action, higher doses of cisatracurium (4×ED_95_ and 6×ED_95_) showed statistically significant longer duration of action than lower doses of cisatracurium and the atracurium (2×ED_95_) [Table 4].

**Table 4:** Neuromuscular blockade after administration of atracurium and cisatracurium

	Onset time (Time to maximum suppression of T1%) (min)	Duration of action (25% recovery T1%) (min)
Atracurium group (n=16) 2×ED_95_	3.24 ± 0.55	44.4 ± 4.13
Cisatracurium group (n=16) 2×ED_95_	4.37 ± 0.46 *	43.6 ± 4.15
Cisatracurium group (n=16) 4×ED_95_	2.9 ± 1.4 #	65.5 ± 10.5 *#
Cisatracurium group (n=16) 6×ED_95_	2 ± 1.2 *#	78.4 ± 8.6 *#

* Statistically significant difference versus 2×ED95 dose of atracurium (*P*-value < 0.05);

# Statistically significant difference versus 2×ED95 dose of cisatracurium (*P*-value < 0.05)

Only 6×ED95 dose of cisatracurium was statistically significant versus the atracurium dose with higher percentages of patients with excellent condition of intubation. 4×ED_95_ and 6×ED_95_ doses of cisatracurium were significantly better than 2×ED_95_ dose of cisatracurium. No one of the studied patients in the four groups been reported as not possible intubation. Assessment of vocal cords, 2×ED_95_ dose of atracurium and 2×ED_95_ and 4×ED_95_ doses of cisatracurium were similar while 6×ED_95_ dose of cisatracurium was significantly better than atracurium and 2×ED_95_ dose of cisatracurium [Table 5].

**Table 5:** Condition of intubation and vocal cords assessment as recoded after 2 minutes of administration of atracurium and cisatracurium

			Atracurium group (n=16) 2×ED_95_	Cisatracurium group (n=16) 2×ED_95_	Cisatracurium group (n=16) 4×ED_95_	Cisatracurium group (n=16) 6×ED_95_
Vocal cords	Open	N (%)	9 (56.25)	6 (37.5)	11 (68.75)	16 *# (100)
	Abducted	N (%)	5 (31.25)	8 (50)	5 (31.25)	0 (0)
	Adducted	N (%)	2 (12.5)	2 (12.5)	0 (0)	0 (0)
Condition of intubation	Excellent	N (%)	6 (37.5)	2 (12.5)	10 # (62.5)	13 #* (81.25)
	Good	N (%)	8 (50)	9 (56.25)	5 (31.25)	3 (18.75)
	Poor	N (%)	2 (12.5)	5 (31.25)	1 (6.25)	0 (0)
	Not possible	N (%)	0 (0)	0 (0)	0 (0)	0 (0)

* Statistically significant difference versus 2×ED_95_ dose of atracurium (*P*-value < 0.05);

# Statistically significant difference versus 2×ED_95_ dose of cisatracurium (*P*-value < 0.05);

Figures in parenthesis are in percentage

No signs of histamine release were noted with any doses of cisatracurium, while it was noted with atracurium (2 cases; 1 case showed flush and the other case showed erythema).

## DISCUSSION

All patients were assessed for hemodynamic state (heart rate, blood pressure), onset time, duration of action, and signs of histamine release clinically, condition of intubations, and vocal cords assessment.

The four groups of the study were matched regarding patients’ age and sex.

Hemodynamic stability for both heart rate and mean arterial blood pressure were more evident among higher doses of cisatracurium (4×ED_95_, 6×ED_95_).

There was a statistically significant increase in HR, MABP postintubation 120 s postinjection of the muscle relaxant when compared to baseline and postinjection of 2×ED_95_ dose of atracurium in group 1 and the same dose of cisatracurium in group 2 because of stress intubation and the patients were not fully relaxed. However, changes in HR and MABP 5-20 minutes later were not statistically significant with administration of 4×ED_95_ and 6×ED_95_ doses of cisatracurium in groups 3 and 4, respectively. Lien **et al**.,[9] and Basta *et al*.,[10] concluded that the maximal MABP and HR changes of patients receiving cisatracurium were small and similar to those observed in patients receiving two times the ED_95_ of atracurium. In his study no patient developed a decrease in blood pressure >20% or an increase in heart rate >20% that was attributable to muscle relaxant administration. Signs of histamine release were shown in one patient in this study in the form of transient facial flushing after the administration of atracurium; however, this patient did not experience hypotension or tachycardia.

The onset time was determined as the interval from the end of muscle relaxant injection until the onset of the maximal suppression of T_1_ and the duration of action of the muscle relaxant was defined as time from disappearance of TOF stimulation till 25% recovery of T_1_.

2×ED_95_ dose of atracurium had more rapid onset of action (with statistical significance) than the equivalent dose of cisatracurium (2×ED_95_). But higher doses of cisatracurium (4×ED_95_ and 6×ED_95_) were found to be statistically significant more rapid onset of action and longer duration of action than 2×ED_95_ dose of both atracurium and cisatracurium. Bluestein and colleagues,[11] studied 80 ASA physical status I or II, 18 – 70 years of age whom were randomly assigned to four groups (A-D). Group A received cisatracurium 0.1 mg/kg (2×ED_95_), group B received atracurium 0.5 mg/kg (2×ED_95_). Patients in group C and group D were treated with cisatracurium 0.2 mg/kg (4×ED_95_) and 0.15 mg/kg (3×ED_95_), respectively. They assessed the mean time of onset, mean time of clinically effective duration, and condition of intubation. As regarding the mean time of onset and mean time of clinically effective duration there results were in accordance with ours. They reported that increasing the initial dose of cisatracurium (from 0.1 to 0.15 and 0.2 mg/kg), decreased the mean time of onset (from 4.6 to 3.4 and 2.8 min, respectively) and increased the mean time of clinically effective duration (45 to 55 and 61 min, respectively). Mellinghoff *et al*.,[3] studied 80 patients randomized to receive either cisatracurium (n=40) or atracurium (n=20) and compared the time course of neuromuscular block. Results obtained by Mellinghoff *et al*.,[3] were similar to our results. They estimated that onset times were 3.1±1.0 min with cisatracurium and 2.3±1.1 min with atracurium (*P*=0.008). After the infusion, the spontaneous recovery intervals were 25-75% of 18±11 min and 18±8 min for cisatracurium and atracurium (*P*=0.896) [Table 6].

**Table 6:** Histamine release among the studied patients

		Atracurium group (n=16) 2×ED_95_	Cisatracurium group (n=16) 2×ED_95_	Cisatracurium group (n=16) 4×ED_95_	Cisatracurium group (n=16) 6×ED_95_	*P*-value
Flush	N (%)	1 (6.5)	0 (0)	0 (0)	0 (0)	0.5
Erythema	N (%)	1 (6.5)	0 (0)	0 (0)	0 (0)	(NS)
Wheals	N (%)	0 (0)	0 (0)	0 (0)	0 (0)	

NS - No statistically significant difference; Figures in parenthesis are in percentage

As regards the condition of intubation in our study, it was estimated that only 6×ED_95_ dose of cisatracurium showed a statistically significant difference versus the atracurium dose with excellent condition of intubation. 4×ED_95_ and 6×ED_95_ doses of cisatracurium were significantly better than 2×ED_95_ dose of cisatracurium. There was not any case of not possible intubation among the four studied groups. The assessment of vocal cords, 2×ED_95_ dose of atracurium, and 2×ED_95_ dose of cisatracurium were similar, while 4×ED_95_ and 6×ED_95_ doses of cisatracurium were significantly better than atracurium and 2×ED_95_ dose of cisatracurium. Results found by Bluestein *et al*.,[11] were consistent with our results. They reported that intubation conditions were good or excellent in over 90% of patients in all treatment groups (2 min after approximately 2×ED_95_ doses of cisatracurium or atracurium and 1.5 min after 3× and 4×ED_95_ doses of cisatracurium).

Mandal[12] conducted a study in 60 adult patients of either sex, belonging to physical status ASA grade I or 11 to find out the minimum possible dose of cisatracurium for achieving excellent to good intubating conditions within 90 s of its administration under general anesthesia.

Patients were divided into three groups according to their dosage schedule. After induction of anesthesia with the standardized method, group I (n=20) received 0.15 mg/kg, group II (n=20) received 0.20 mg/kg, and group III (n=20) received 0.25 mg/kg of cisatracurium. For each group laryngoscopy and intubation was tried at either 75 or 90s, thereby patients were further divided into six subgroups. Subgroup ‘a’ denotes the procedure at 75s and, ‘b’ denotes at 90s. For grading the intubating conditions, the ease of laryngoscopy, the position or movement of the vocal cords and the degree of coughing were evaluated. Excellent to good intubating conditions could be achieved only in group IIb (0.20 mg/kg at 90 s) and both subgroups (0.25 mg/kg at 7 5s and 90 s) in group III patients. Hence the minimum dose required to achieve excellent to good intubating conditions with cisatracurium is 0.20 mg/kg at 90 s after its administration. The adequacy of conditions for tracheal intubation is a function of several factors, such as the depth of anesthesia at the time of the intubation attempt and the level of neuromuscular block at the time of attempt.[13]

One of two intubating doses of cisatracurium may be chosen based on the desired time of intubation and the anticipated length of surgery. Doses of 0.15 mg/ kg (3×ED_95_) and 0.2 mg/kg (4×ED_95_) of cisatracurium, as components of a propofol /nitrous oxide/oxygen induction intubation technique, may produce generally good or excellent conditions of intubation in 2.0 and 1.5 min, respectively.[14]

The cisatracurium dose of 0.15 mg/kg (3×ED_95_) is higher than the dose of atracurium 0.5 mg/kg (2×ED_95_) required to produce clinically acceptable intubation conditions after 120 s. However, this dose of cisatracurium still provides neuromuscular block of intermediate duration and following rapid administration not associated with histamine-mediated cardiovascular effects.[13]

No signs of histamine release were noted in any doses of cisatracurium while it was noted with atracurium (2 cases; 1 case showed flush and the other case showed erythema).

Signs of histamine release were shown in two patients with administration of 2×ED_95_ dose of atracurium in group 1, one patient in the form of flushing at the site of injection in the ventral aspect of the forearm but the patient did not experience hypotension or tachycardia and the other patient had shown transient erythema after the administration of atracurium and tachycardia only (an increase in heart rate<20% of base line) without any decrease in blood pressure.

As a benzylisoquinoline, atracurium has the potential for release of histamine. The syndrome becomes clinically evident when doses of 0.5 mg/kg (two times ED_95_) or more are injected rapidly.[10]

When plasma histamine levels increase to over 1000 pg/ml, a transient decrease in blood pressure, together with facial erythema, may be noted. The phenomenon of histamine release may be shifted to the right by slower injection from 30 to 60 s.[10]

Combined H_1_ and H_2_ receptors blockade effectively prevents the cardiovascular manifestation of histamine release. Hosking *et al*., have treated patients with diphenylhydramine 1 mg/kg and cimetidine 4 mg/kg was given intravenously 30 min before giving a very large dose of atracurium (1.5 mg /kg or six times ED_95_), and the atracurium-induced decrease in mean arterial blood pressure was reduced to 30 mmHg (37%below baseline) in treated patients.[15] Despite a 10 to 20-folds increase in plasma histamine levels atracurium is non-vagolytic and does not block autonomic ganglia.[16]

We can conclude that at the same dose (2×ED_95_ dose) atracurium is more effective neuromuscular blocking agent than cisatracurium, while higher doses of cisatracurium provide more effective, more rapid neuromuscular blocking with longer duration of action, stable hemodynamic status, and no associated signs of histamine release clinically.

## References

[1] Kleinman W, Nitti GJ, Nitti JT, Raya J, Morgan GE, Mikhail MS, Murray MJ (2006). Neuromuscular blocking agents. Clinical anesthesiology.

[2] Kleinman W, Nitti GJ, Nitti JT, Raya J, Morgan GE, Mikhail MS, Murray MJ (2006). Neuromuscular blocking agents. Clinical anesthesiology.

[3] Mellinghoff H, Radbrush L, Diefenbach C, Buzello W (1996). A comparison of cisatracurium and atracurium: onset of neuromuscular block after bolus injection and recovery after subsequent infusion. Anaesth Analg.

[4] Kirov K, Motamed C, Decailliot F, Behforouz N, Duvaldestin P (2004). Comparison of the neuromuscular blocking effect of cisatracurium and atracurium on the larynx and the adductor pollicis. Acta Anaesth Scand.

[5] Nogueira CM, Sudo GZ, Sudo RT (2010). Hemodynamic effects of atracurium and cisatracurium and the use of diphenhydramine and cimetidine. Rev BrasAnestesiol.

[6] Hermann M, Lukas R, Christoph D, Walter B (1996). A comparison of cisatracurium and atracurium: onset of neuromuscular block after bolus injection and recovery after subsequent infusion. Anesth Analg.

[7] Goldberg M, Larijani G, Azad S (1989). Comparison of tracheal intubation conditions and neuromuscular blocking profiles after intubating dose of mivacurium chloride or succinylcholine in surgical outpatients. Anesth Analg.

[8] Doenicke A, Moss J, Lorenz W, Gottardis M (1994). Are hypotension and rash after atracurium caused by histamine release?. Anesth Analg.

[9] Lien CA, Belmont MR, Abalos A (1995). The cardiovascular effects and histamine-releasing properties of 51W89 in patients receiving nitrous oxide / opioid / barbiturate anesthesia. Anesthesiol.

[10] Basta SJ, Ali HH, Savarese JJ (1992). Clinical pharmacology of atracurium besylate: a new nondepolarizing muscle relaxant. Anaesth Analg.

[11] Bluestein LS, Stinson LW, Lennon RL, Wilson RM (1996). Evaluation of cisatracurium, anew neuromuscular blocking agent for tracheal intubation. CAN J ANAESTH.

[12] Mandal P (2002). Intubating Conditions after Cisatracurium Administration-A Dose Response Study in Adults. J Anaesth Clin Pharmacol.

[13] Belmont MR, Lien CA, Quessy S (1995). The clinical neuromuscular pharmacology of 51W89 in patients receiving nitrous oxide/opioid/barbiturate anesthesia. Anesthesiol.

[14] Schmautz E, Deriaz H, Vrillon M (1994). Evaluation of 51W89 for endotracheal intubation in surgical patients during N_2_O/O_2_/propofol anesthesia. Anesthesiol.

[15] Hosking MP, Lennon RL, Gronert GA (1988). Combined H1 and H2 receptor blockade attenuates the cardiovascular effects of high dose atracurium for rapid sequence endotracheal intubation. Anaesth Analg.

[16] Hughes R, Chapple DJ (1981). The pharmacology of atracurium a new competitive blocking agent. Br J Anaesth.

